# Extracorporeal Cytokine Hemadsorption for the Treatment of Severe Steroid Refractory Cytokine Release Syndrome Secondary to T‐Cell Immunotherapy for B‐Cell Malignancies: A Case Series

**DOI:** 10.1002/hon.70210

**Published:** 2026-06-22

**Authors:** Gianluca Cavallaro, Silvia Ferrari, Anna Pollastri, Elena Di Credico, Cristian Meli, Monica Rondi, Alessandro Putelli, Federico Lussana, Anna Grassi, Alessandro Rambaldi, Tino Martino Valetti, Ivano Riva, Giuseppe Gritti

**Affiliations:** ^1^ Hematology and Bone Marrow Transplant Unit ASST Papa Giovanni XXIII Bergamo Italy; ^2^ Intensive Care Units ASST Papa Giovanni XXIII Bergamo Italy; ^3^ Foundation of Clinical Research‐FROM Azienda Socio‐Sanitaria Territoriale Papa Giovanni XXIII Bergamo Italy

**Keywords:** B‐cell acute lymphoblastic leukemia, B‐cell non‐hodgkin lymphoma, bispecific antibodies, CAR t‐cell therapy, cytokine release syndrome

## Conflicts of Interest

G.G. has participated in advisory boards for Abbvie, Roche, Takeda, Kite‐Gilead, Ideogen, Genmab and receives research funding from Ideogen, outside the submitted work. I.R. reports travel support from Aferetica. All other authors declare no competing interests.

To the Editor,

Cytokine release syndrome (CRS) management follows the ASTCT guidelines [1], but steroid‐refractory CRS is an increasingly recognised unmet clinical need [2]. CytoSorb (Cytosorbent, USA) is a hemoadsorption device containing polymer beads designed to irreversibly remove cytokines. It is currently used for septic shock and other conditions associated with elevated cytokine levels [3, 4, 5], providing a strong rationale for its application in severe steroid‐refractory CRS. We present a case series of four patients, who developed grade 4 steroid‐refractory CRS following treatment with either CD19‐CAR‐T cells or CD3xCD20 bispecific antibody (BsAb), which were managed with cytokine adsorption therapy. In two patients the rapid reduction of plasmatic inflammatory cytokines with CytoSorb treatment was documented.

Case 1 B‐cell acute lymphoblastic leukemia treated with CD19 CAR T‐cells.

**FIGURE 1:**
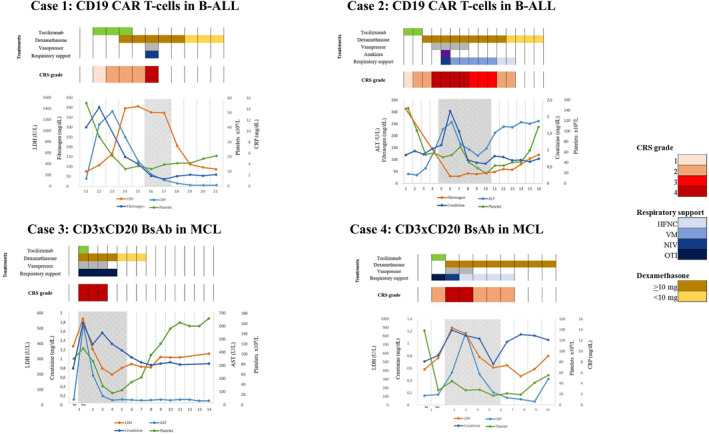
Summary of toxicity, management and key laboratory findings. Summary of the key laboratory findings, CRS grading and therapeutic interventions on a daily basis for each case. Dotted rectangle indicates treatment period with Cytosorb.

**FIGURE 2:**
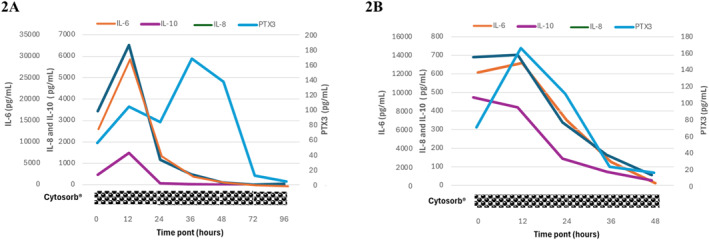
Plasma levels of inflammatory cytokines during CytoSorb treatment. (A) Case 1; (B) Case 2.

A 32‐year‐old woman with relapsed B‐cell acute lymphoblastic leukemia (B‐ALL) relapsed was treated with CD19‐CAR T‐cell therapy in a clinical trial. At the time of infusion, bone marrow assessment showed 40% disease involvement. Eighteen days after CAR‐T infusion, she developed grade 2 CRS, requiring three doses of tocilizumab (8 mg/Kg) and dexamethasone 10 mg every 8 h. The CRS progressed to grade 4 within three days, for which she was admitted to ICU, where norepinephrine (0.05 μg/kg/min) was initiated and continuous veno‐venous hemodialysis (CVVHD) with Cytosorb was implemented. Cytokine hemoadsorption was maintained for 48 h, leading to vasopressor discontinuation and diuresis recovery, allowing for dexamethasone tapering 24 h after the last hemoadsorption cycle. The patient was discharged from the ICU after 4 days. CAR‐T cell expansion was robust, peaking at 347.9 cells x10^6^/L on day 28 post‐infusion, with a continuous increase observed even during cytokine hemoadsorption. Figure 1 summarize the key laboratory findings, CRS grading and therapeutic interventions on a daily basis for each case. Plasma levels of inflammatory cytokines, including IL‐6, IL‐8, and IL‐10, were sequentially measured during CytoSorb treatment using BD CBA Human Inflammatory Cytokines Kit (BD Biosciences, San Jose, CA, USA), along with pentraxin 3 (PTX3). A progressive decrease of all the inflammatory cytokines was observed, correlating with the clinical resolution of CRS (Figure 2A). Despite achieving a complete remission (CR), disease relapsed 6 months after CAR‐T; the patient received inotuzumab and a second allogeneic transplant, but subsequently relapsed and died of disease progression.

Case 2 B‐cell acute lymphoblastic leukemia treated with CD19 CAR T‐cells.

A 25‐year‐old woman with relapsed B‐cell acute lymphoblastic leukemia (B‐ALL) received CD19‐CAR T‐cell (tisagenlecleucel). At the time of infusion, bone marrow assessment showed 20% leukemic infiltration. By day 2 after CAR‐T infusion, she developed grade 2 CRS, prompting treatment with 2 doses of tocilizumab (8 mg/Kg), along with dexamethasone 10 mg every 8 h. On day 6, due to sustained fever and clinical deterioration, anakinra (100 mg SC) was added. The patient was transferred to the ICU due to liver failure, coagulopathy, hypotension and oligo‐anuria. She required non‐invasive ventilation and vasopressors support, including norepinephrine (0.11 μg/kg/min) and adrenaline (0.04 μg/kg/min). CVVHDF was initiated along with Cytosorb hemoadsorption therapy. During the first cycle of extracorporeal hemoadsorption, a rapid improvement in clinical condition and vital signs was observed, allowing for vasopressors discontinuation, with diuresis resumption. Dexamethasone was tapered 72 h after the last hemoadsorption cycle. She underwent five cycles of blood purification with Cytosorb and was discharged from the ICU after 1 week. CRS manifestations coincided with robust CAR‐T cell expansion, peaking at 5021 cells x10^6^/L on day 6 post‐infusion. Plasma levels of inflammatory cytokines initially rose but progressively declined with CytoSorb treatment, correlating with both clinical and laboratory improvement (Figure 2B). At the last follow‐up, she is currently alive and in complete remission.

Case 3 Mantle cell lymphoma treated with CD3xCD20 bispecific antibody.

A 55‐year‐old man with relapsed/refractory mantle cell lymphoma was treated with a CD3xCD20 BsAb in a clinical trial. After 24 h, he experienced grade 2 CRS with fever, hypotension, and mild respiratory failure requiring low flow oxygen. Tocilizumab (8 mg/Kg) and methylprednisolone (100 mg) were administered. Hypotension remained refractory to fluid resuscitation and respiratory failure escalated to severe acute respiratory distress syndrome (ARDS), accompanied by oligoanuria, consistent with multi‐organ failure (MOF). He was transferred to the ICU, where he required orotracheal intubation, mechanical ventilation and norepinephrine support (0.3 mcg/kg/min). A second dose of tocilizumab (8 mg/Kg) and dexamethasone (10 mg four times daily) were administered; MOF showed no signs of improvement. CVVHDF with a CytoSorb filter was then initiated. A rapid hemodynamic response was observed, allowing for vasopressor discontinuation within the first 24 h and subsequent dexamethasone tapering. Clinical improvement continued, leading to the cessation of CytoSorb after 12 h. By the second day of ICU admission, the patient was extubated and vasopressor support was interrupted the following day, with diuresis resumption. He was discharged from the ICU after 4 days, with complete clinical and laboratory resolution of MOF and no further signs of CRS. After achieving CR, the patient had an isolated central nervous system relapse and he did not respond to chemotherapy.

Case 4 Mantle cell lymphoma treated with CD3xCD20 bispecific antibody.

A 64‐year‐old man with blastoid mantle cell lymphoma, refractory to six prior lines of therapy, was treated with a CD3xCD20 BsAb in a clinical trial. Twelve hours after the first infusion, he developed grade 2 cytokine release syndrome (CRS), which rapidly escalated to grade 4, presenting with ARDS and severe hemodynamic impairment. He was treated with tocilizumab (8 mg/Kg) and dexamethasone (10 mg) and subsequently admitted to the ICU. Due to persistent anuria, CVVHD with CytoSorb hemoadsorption was initiated. Despite these interventions, hemodynamic instability worsened, requiring norepinephrine infusion (0.18 mcg/kg/min). Over the following days, the patient showed progressive clinical improvement. Vasopressor support was discontinued after 4 days, fever resolved, diuresis resumed within the first 24 h of treatment and dexamethasone was tapered and stopped afterward. Hemodialysis was stopped on ICU‐day five. Eight months after achieving CR, the patient experienced disease relapse; he received CAR‐T, with a short duration CR and subsequently died of disease progression.

In severe CRS cases, targeting a single cytokine or using high‐dose corticosteroids may not be sufficient to prevent rapid progression to a life‐threatening condition [8]. A recent small case series described the use of emapalumab, ruxolitinib, and low‐dose etoposide in immune effector cell‐associated hemophagocytic lymphohistiocytosis‐like syndrome [9]. However, many of these agents have the potential to impair the effectiveness of BsAb or CAR T‐cell therapy.

Advanced CRS is characterized by a cytokine storm, due to the complex interaction between the CAR‐T, cancer cells and macrophages. CytoSorb may represent a promising strategy to rapidly and non‐selectively remove pro‐inflammatory mediators up to the size of 60 kDa, sparing larger molecules, such as BsAb, whose pharmacokinetics is not hampered by hemadsorption. Likewise, cytokines removal does not affect CAR‐T in vivo expansion, as showed in our patients.

Literature data on CytoSorb for refractory CRS are limited to single case reports, showing that cytokines hemadsorption can lead to rapid clinical improvement within few days from treatment start, allowing for reduction of life‐supporting care [6]; in a case report, plasmatic cytokines clearance was also documented [7], as for our patients.

In our experience, we consider CytoSorb for patients with steroid‐refractory grade 4 CRS/ICANS admitted to Intensive Care Unit. The treatment is performed with Continuous Renal Replacement Therapies (CRRT) and the cartridge is changed every 12 hours until clear signs of hemodynamic, respiratory and organs disfunction improvement.

With the limitation of a small case series, we showed that CytoSorb treatment is both feasible and safe and could serve as an adjunctive therapy in severe cases of CRS, warranting further evaluation in clinical practice in prospective and multicenter studies, including possible comparison between patients receiving hemadsorption or standard‐of‐care treatments. In this regard, there are few prospective trials investigating the role of Cytosorb in severe CRS cases and other hyperinflammatory syndromes (clinicaltrials. Gov; NCT04048434, NCT05146336).

## Author Contributions

G.C., G.G. and I.R. conceived and designed the study; G.C., A.P., E.D.C., I.R., G.G. collected clinical data and wrote the manuscript; G.C., S.F., A.P., E.D.C., M.R., A.P., F.L., A.G., I.R., G.G. treated the patients; C.M. performed the measurement of plasmatic inflammatory cytokines; All authors contributed to data interpretation, critical review and final approval of the manuscript.

## Funding

This work was supported by Fondazione AIRC per la Ricerca sul Cancro (AIRC) AIRC 5X1000 under Grant AIRC‐ISM ID 21147 and the Associazione Italiana Lotta alla Leucemia, Linfoma e Mieloma (AIL) Sezione Bergamo.

## Data Availability

The data that support the findings of this study are available from the corresponding author upon reasonable request.
